# On the Role of Histamine Receptors in the Regulation of Circadian Rhythms

**DOI:** 10.1371/journal.pone.0144694

**Published:** 2015-12-10

**Authors:** Stanislav V. Rozov, Tarja Porkka-Heiskanen, Pertti Panula

**Affiliations:** 1 Neuroscience Center and Department of Anatomy, Faculty of Medicine, University of Helsinki, Helsinki, Finland; 2 Department of Physiology, Faculty of Medicine, University of Helsinki, Helsinki, Finland; McGill University, CANADA

## Abstract

Several lines of evidence suggest a regulatory role of histamine in circadian rhythms, but little is known about signaling pathways that would be involved in such a putative role. The aim of this study was to examine whether histamine mediates its effects on the circadian system through *Hrh1* or *Hrh3* receptors. We assessed both diurnal and free-running locomotor activity rhythms of *Hrh1* ^-/-^ and *Hrh3* ^-/-^ mice. We also determined the expression of *Per1*, *Per2* and *Bmal1* genes in the suprachiasmatic nuclei, several areas of the cerebral cortex and striatum under symmetric 24 h light-dark cycle at zeitgeber times 14 and 6 by using radioactive *in situ* hybridization. We found no differences between *Hrh1* ^*-/-*^ and wild type mice in the length, amplitude and mesor of diurnal and free-running activity rhythms as well as in expression of *Per1*, *Per2* and *Bmal1* genes in any of the examined brain structures. The amplitude of free-running activity rhythm of the *Hrh3* ^*-/-*^ mice was significantly flattened, whereas the expression of the clock genes in *Hrh3* ^*-/-*^ mice was similar to the wild type animals in all of the assessed brain structures. Therefore, the knockout of *Hrh1* receptor had no effects on the circadian rhythm of spontaneous locomotion, and a knockout of *Hrh3* receptor caused a substantial reduction of free-running activity rhythm amplitude, but none of these knockout models affected the expression patterns of the core clock genes in any of the studied brain structures.

## Introduction

Circadian rhythms are an adaptation phenomenon that has been described for virtually all living organisms. In mammals it is maintained by the master circadian oscillator in the suprachiasmatic nuclei of the hypothalamus (SCN; [1,2]) and entrained by external timed cues, such as light and feeding. The master clock then orchestrates a network of semiautonomous and slave oscillators in other brain regions (for review see [3]). At the molecular level, the oscillator consists of coordinated interlocking autoregulatory transcription and translation loops of several genes, including *Period* (*Per1*, *Per2*), *Cryptochrome* (*Cry1*, *Cry2*), *Bmal1*, *Rev-erbα* and *Clock* [4,5]. Although the expression of these genes in the SCN is essential to control biological rhythms, their expression in the cerebral cortex is associated with a state of alertness rather than the phase of the master clock [6–9].

Histaminergic neurons localize in the posterior hypothalamus of vertebrates [10,11] and these comprise a significant part of the ascending arousal system (reviewed in [12]), which, among other functions, regulates cortical arousal and maintains an alertness state [13]. In mammals histaminergic neurons abundantly innervate cerebral cortex, brain regions involved in regulation of sleep-wake cycle, such as pontine tegmentum, preoptic area, basal forebrain [14], and the SCN [15]. Mice lacking the key histamine synthesizing enzyme histidine decarboxylase (*Hdc*) exhibit abnormal circadian rhythms of locomotor activity and disappearance of circadian periods of *Per1* and *Per2* genes expression in the striatum and cerebral cortex [16]. Pharmacological inhibition of *Hdc* in rats causes significant attenuation of the amplitude of rhythms of adrenocorticotropic hormone and corticosterone [17].

In order to understand which histamine receptors mediate changes in free-running activity and clock genes expression, mentioned above, we analyzed locomotor rhythms of *Hrh1* ^-/-^ and *Hrh3* ^-/-^ mice under normal 12h light /12h dark (LD12/12) cycle and constant darkness (DD), and the expression of *Per1*, *Per2* and *Bmal1* genes in the SCN, several cortical regions and the striatum.

## Materials and Methods

### Animals

Seven- to 10-week-old mice of both sexes of the following genotypes: C57BL/6JCrl *Hrh1* ^-/-^, and C57BL/6JCrl *Hrh3* ^-/-^ were used along with a background strain as the control group. The *Hrh1* ^-/-^ mice [18] were received from the Riken Research Center for Allergy and Immunology and maintained in local animal facility on C57BL/6JCrl genetic background. The *Hrh3* ^-/-^ mice were supplied by Johnson and Johnson Pharmaceutical Research and Development, LLC, La Jolla, CA. The *Hrh3* ^-/-^ mice had been bred and maintained by the Jackson Laboratory by selective backcrossing with C57BL/6J mice for at least 10 generations to show at least 99.5% identity with the C57BL/6J strain [19]. The mice were then delivered to local animal facility. The mice of both genotypes were back-crossed with the background strain every 6–7 generations. The animals were taken from heterozygous non-brother-sister matings, thus littermates were used for the study. Animals were housed individually in plastic Macrolon cages (20x30 cm) with an open top in a sound-proof room (12 cages per room) at 22.5 ± 1°C; standard food pellets (Scanbur, Sollentuna, Sweden) and water were provided ad libitum. The illumination at the bottom of the cages during the photophase was 150±20 lux, and complete darkness during scotophase. The animals were kept under LD12/12 for one week, then for two weeks under (DD) and afterwards kept under LD12/12 for one week. All experiments were performed in compliance with the Finnish Act on the Use of Animals for Experimental Purposes. The protocol of the study was approved by the Animal Experiment Committee of the State Provincial Office of Southern Finland. All experiments were carried out in accordance with the European Communities Council Directive of 24 November 1986 (86/609/EEC), and followed the Guidelines laid down by the NIH in the USA regarding the care and use of animals for experimental procedures. The total number of animals used in this study was 62.

### Chemicals

The chemical reagents used in the study were as follows: deoxyadenosine 5′-triphosphate, [α-^33^P] (NEG312H; NEN Research Products, PerkinElmer, Waltham, MS, USA); CaCl_2_, NaCl, KCl (Merck, Whitehouse Station, NJ, USA); aminoguanidine hydrochloride, Denhardt's solution, dithiothreitol, N-lauroylsarcosine, polyethylene glycol 300, β-mercaptoethanol, phenylmethanesulfonyl fluoride, S-adenosyl-methionine (Sigma, St Louis, MO, USA); MgCl_2_ and (Riedel-deHaёn, Seelze, Germany); formamide (Amresco, Solon, OH, USA); RNA (Roche, Basel, Switzerland); and dextran sulphate (Amersham, Amersham, UK).

### Locomotor activity assessment

Each cage was equipped with a video camera model CAMZWMBLAH2N (Velleman, Gavere, Belgium) equipped with an infra-red light source to enable recording in the darkness. The video stream was captured and recorded continuously with GeoVision surveillance software (GeoVision, Taiwan). The recorded video data were prepared for tracking with Virtualdub 1.9.2 (www.virtualdub.org), and tracking was then performed by subjecting the video data to analysis using the Ethovision 3.1 software package (Noldus Software, Wageningen, Netherlands). The average distance moved was calculated in 1-min time bins.

### Radioactive in situ hybridization

The groups of mice were euthanized by decapitation at ZT6 and ZT14 upon completion of the behavioral study. The brains were removed, rapidly frozen by immersion into cooled isopentane (-45°C), and then subsequently stored at −80°C. Just before sectioning frozen brains were covered with M1 embedding matrix (ThermoScientific, Calamazoo, MI, USA) then coronal 20-μm sections were cut using a Leica CM3050S cryostat (Leica, Wetzlar, Germany), and then mounted onto SuperFrost slides (ThermoScientific, Portsmouth, USA). The sections were stored at −20°C until analysis.

The expression levels of *Per1*, *Per2* and *Bmal1* mRNA were analyzed in the striatum, the motor, somatosensory and cingulate areas of cerebral cortex and in the SCN. The procedure that we applied had been described previously [20], and was used in the present study but with minor modifications [21]. DNA oligoprobes used in our study were the same as those described by Abe et al., [16] and had the following sequences:

*Per1*: 5'-TGCTTGTATGGCTGCTCTGACTGCTGCGGGTGATGCTGGCTGAGG-3'

*Per2*: 5'-GCTCCTTCAGGGTCCTTATCAGTTCTTTGTGTGCGTCAGCTTTGG-3'

*Bmal1*: 5'-GCCATTGCTGCCTCATCGTTACTGGGACTACTTGATCCTTGGTCG-3'

The oligoprobes were labeled with deoxyadenosine 5′-triphosphate, [α-^33^P] at their 3′-ends by using terminal deoxynucleotide transferase (Promega, Madison, WI, USA), according to the manufacturer's instructions. Non-incorporated nucleotides were removed by purification with Sephadex G-50 QuickSpin cartridges (Roche). Between six to ten brain sections per mouse were hybridized with either probe at 45°C for 24 h. After a series of high-stringency washes in SSC solution (0.015M sodium citrate, 0.15M NaCl), at 56°C, that removed non-hybridized probe excess, Kodak BioMax films were exposed to the sections and the ^14^C-standards (Amersham). Images of the standards and autoradiograms of the brain sections were acquired in gray-scale (8 bit) settings by a digital camera and analyzed using the MCID 6.0 platform (Imaging Research, St Catharines, Canada). A film background (in gray levels) was measured adjacent to every tissue autoradiogram and for the standard. The mean intensity values (in gray levels) were acquired from tissue autoradiograms corresponding to the striatum and cortical areas and linearized using the standards. The SCN region was defined based on the histology of the brain sections and grain intensity on the film: the lower threshold of intensity window was determined as two standard deviations below the mean pixel intensity of the background area. Only pixels with the intensities exceeding the lower threshold were included in the analysis. Then optical densities of acquired samples and standards were computed as:

$$OD=log_{10}\left(I_{O}/I_{T}\right)$$ (1)

whereby *I*_*o*_ - background intensity, in gray levels; *I*_*T*_ - signal intensity, in gray levels. Therefore, the *OD* values represent the opacity of the film in response to exposure to a radioligand that had been corrected for film background. *OD* values of standards were fitted with one phase exponential association function:

$$y=y_{max}\left(1-e^{-kx}\right)$$ (2)

whereby *y*_max_ - maximum log_10_-intensity, *k* - coefficient of exponential slope. The intensity values of each autoradiogram converted to corresponding uCi/g values by using Eq 2. The resulting values were averaged for each mouse and processed further as described below.

### Data analyses and statistics

Period lengths under LD and DD conditions were determined by taking the locomotor activity time series that was acquired from individual animals and analyze them by using a non-parametric χ^2^-periodogram method [22] in a range of periods from 16 to 32 h at 0.5 h resolution. Maximum *Qp* values were used for a group comparison using the Student’s t-test.

The behavioral data from individual subjects were pooled into 30 min bins to estimate mesors and amplitudes of locomotor activity. Individual tracks were averaged for each bin over the course of experiment to get group-averaged time series, which reduced the within-group variability caused by random between-subjects variation. The track-means were then fitted with cosine curve by the cosinor method [23] using the periodicity that had been identified by χ^2^-periodogram. Afterwards mesor and amplitude parameters of these cosinor models were computed.

We tested the null hypothesis for the absence of differences of mesors and amplitudes between the groups, by using permutation tests with Monte-Carlo sampling. These permutation tests were performed using Matlab 7.5 (Matlab, Nattick, USA) as follows: every iteration subjects were randomly allocated between the groups, followed by group-averaging, and cosinor modelling as described above. The resulting distributions of differences of amplitudes and mesors were used to acquire significance level by the Z-test.

The activity fragmentation was assessed by counting individual activity bouts identified as periods of continuous activity of more than 20 cm/min and separated by at least two minutes of resting.

The analysis of activity fragmentation and gene expression had to account for several issues. First, some experimental groups had unequal variances and numbers of subjects per group. Second, analyses were performed on the same subjects repeatedly, hence producing correlated measures. Third, individual variation for estimated parameters. Therefore, we used the *nlme* library of R software [24] to conduct linear mixed effect analysis of an effects of genotype, time of day and illumination regime on activity fragmentation and also the effect of genotype and time of day on expression of *mPer1*, *mPer2* and *Bmal1* genes in different brain regions, corrected for the constrains mentioned above. The models were specified as follows: analysis of activity fragmentation:

$$y_{iklm}=\mu +\alpha_{k}+\beta_{l}+\chi_{m}+\delta_{lm}+b_{i}+\varepsilon_{iklm}$$ (3)

whereby ***y***_*iklm*_ - response vector of *i* ^*th*^ subject of *k* ^th^ genotype at daytime *l* under regime *m*; *μ* - grand mean; *α*_*k*_ - main effect of genotype; where *k* = 1,2; *β*_*l*_ - main effect of daytime; where *l* = 1,2; *χ*_*m*_ - main effect of regime; where *m* = 1,2; *δ*_*lm*_ - daytime-regime interaction; *b*_*i*_ - subject random effect; *ε*_*iklm*_ - random error; *b*_*i*_~*N*(0,Ψ), *ε~N*(0,*σ* ^2^ *I*) analysis of gene expression:

$$y_{iklm}=\mu +\alpha_{k}+\beta_{l}+\chi_{m}+\delta_{kl}+\phi_{lm}+\gamma_{km}+\eta_{klm}+b_{i}+\varepsilon_{iklm}$$ (4)

whereby y_iklm_ - response vector of i^th^ subject of k^th^ genotype at time l in structure m; *μ* - grand mean; *α*_*k*_ - main effect of genotype; where k = 1,2; *β*_*l*_ - main effect of time; where l = 1,2; *χ*_*m*_ - main effect of structure; where m = 1,…5; *δ*_*kl*_ - genotype-time interaction; *ϕ*_*lm*_ - time-structure interaction; *γ*_*km*_ - genotype-structure interaction; *η*_*klm*_ - genotype-time-structure interaction; b_i_ - subject random effect; *ε*_*iklm*_ - random error; *b*_*i*_~*N*(0,Ψ), *ε~N*(0,*σ* ^2^ *I*)

For the analysis of activity fragmentation, genotype, time of day and illumination regime were chosen as fixed factors and intercepts per subject—as a random effect.

For the gene expression analysis, we chose genotype and time of day as between-subject factors, the brain region—as a within-subject factor, and the intercepts per subject as random effects. The measurements of gene expression were correlated but correlation structure could not be readily deduced, therefore unstructured variance-covariance matrices were specified to account for it. In those cases in which the visual evaluation of plots of residuals revealed heteroscedasticity, a regional weighting of the variances was applied. The research question of these experiments was whether or not the genotype has an effect on the expression of the genes at different time points in a different brain areas. Therefore, p-values were obtained by the likelihood ratio (LR) tests of the full model with the factor “genotype” compared against the model without this factor. The confidence intervals (CI) of estimated compounds, overall fitting of the data set by the model, as well as assumptions of normality and homogeneity of residuals were tested by visual inspection.

## Results

### Effects of *Hrh1* and *Hrh3* knockout on locomotor activity

**Fig 1:**
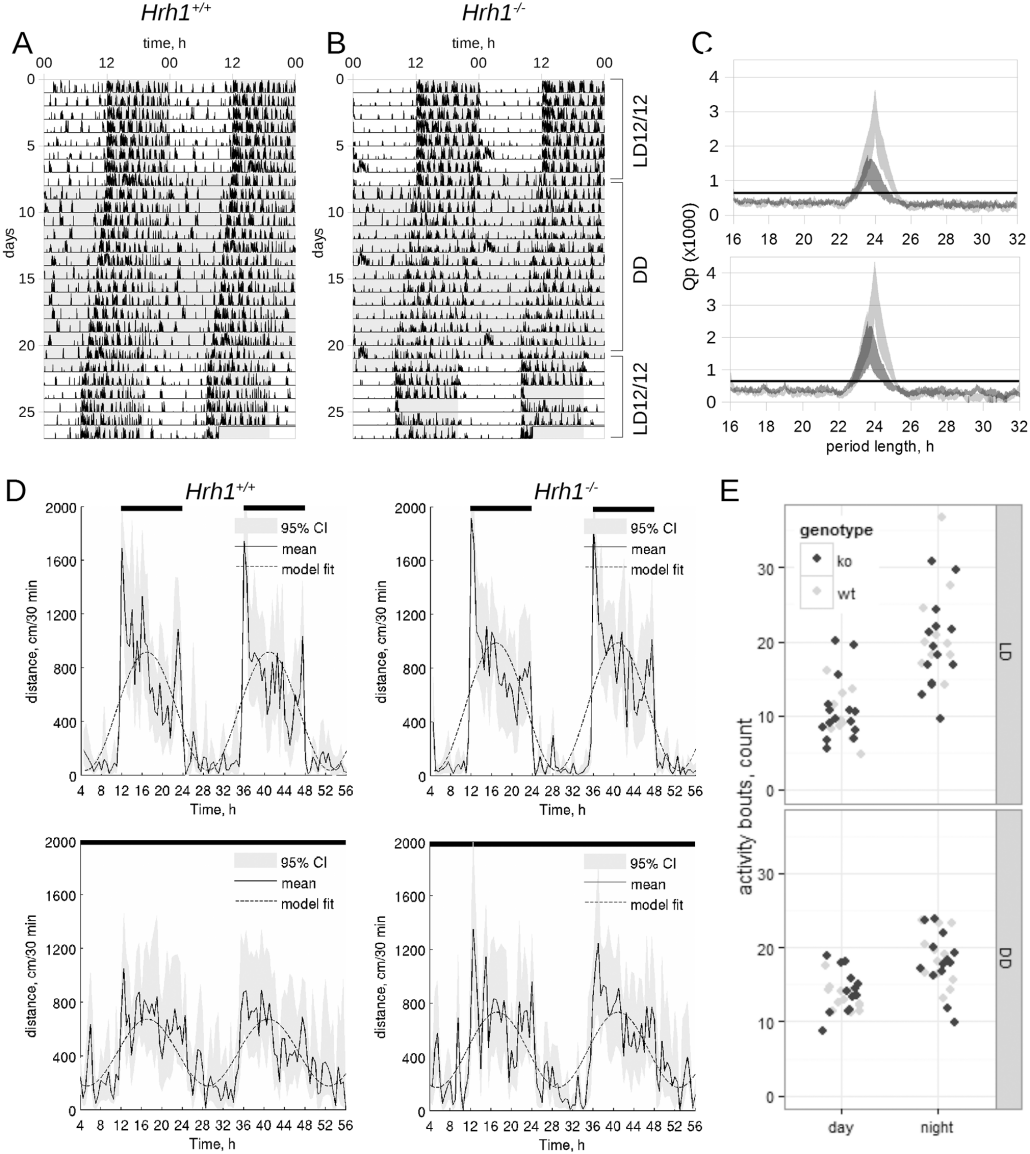
Individual actograms of wild type (A) and *Hrh1* ^*-/-*^ (B) mice. Animals were housed for one week under LD12/12, then in DD for two weeks and finally for one week under LD12/12. The gray area corresponds to darkness. Analysis of locomotor activity by χ^2^-periodogram (C). Periodograms are plotted as 95% CI of means of a wild type (upper panel) and knockout (lower panel) mice under LD12/12 (light gray) or DD (dark gray) conditions, the horizontal solid line represents the *Qp* values at p = 0.01; n = 12 (wild type), 13 (*Hrh1* ^*-/-*^). Cosinor analysis of locomotor activity under LD12/12 (D, upper panel) and DD (D, lower panel) regimes: black solid line show averaged locomotor activity over 48 h, black dashed line represents cosine fit for the averaged activity, light gray area show 95% CI of averaged activity. Analysis of activity fragmentation (E) under LD12/12 (upper panel) and DD (lower panel) regimes of wild type (light gray circles) and *Hrh1* ^*-/-*^ (black circles) animals.

**Fig 2:**
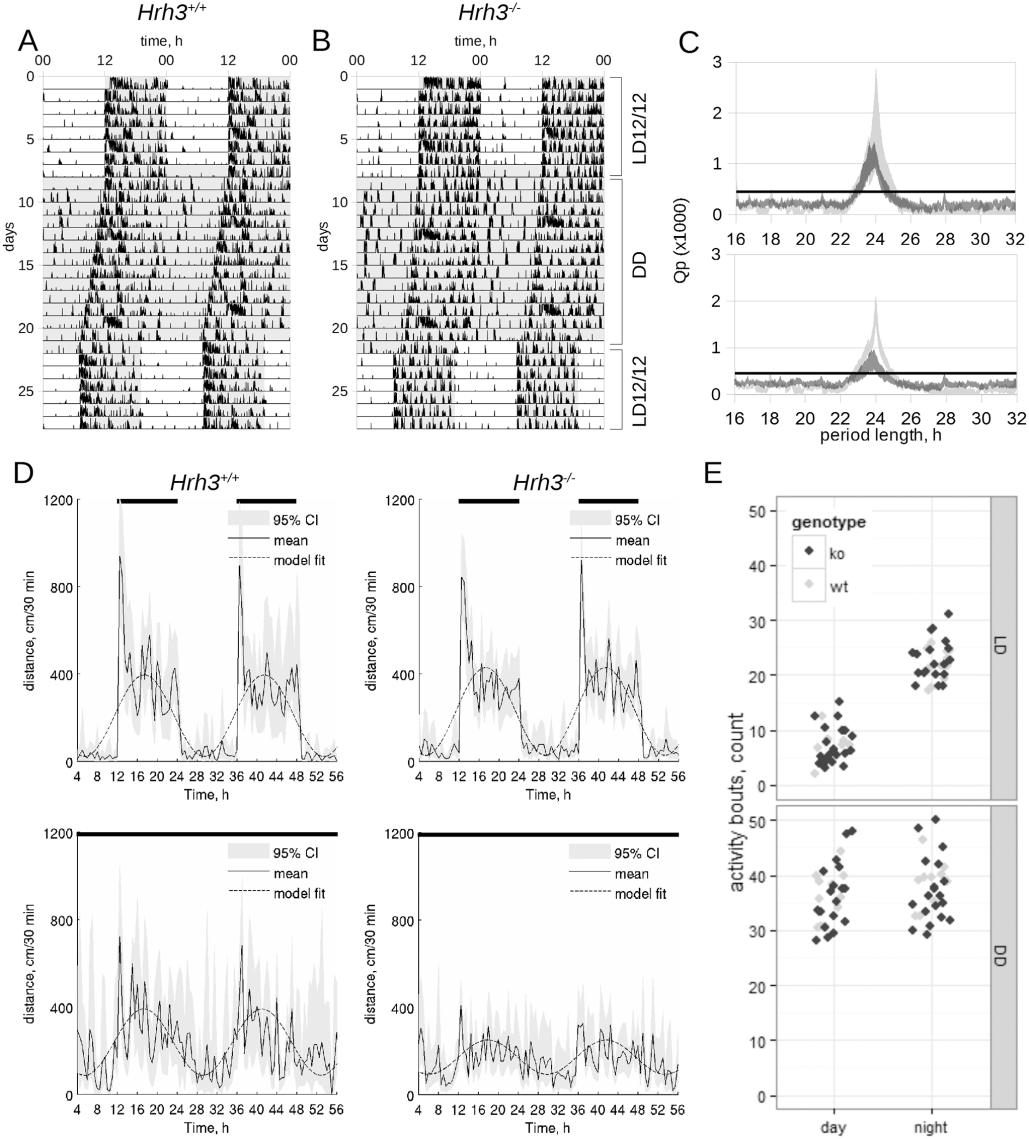
Individual actograms of a wild type (A) and *Hrh3* ^*-/-*^ (B) mice. Animals were housed for one week under LD12/12, then kept in constant darkness for two weeks and then finally for one week under LD12/12. The shaded area corresponds to a darkness period. Analysis of locomotor activity with χ^2^-periodogram (D and E). Periodograms are plotted as 95% CI of means of a wild type (D) and knockout (E) mice under LD12/12 (light gray) or constant darkness (dark gray, DD) conditions, horizontal solid line—*Qp* value at p = 0.01; n = 11 (wild type), 19 (*Hrh3* ^*-/-*^). Cosinor analysis of locomotor activity under LD12/12 (D, upper panel) and DD (D, lower panel) regimes: black solid line shows averaged locomotor activity over 48 h, the black dashed line indicates the cosine fit of the averaged activity, light grey area represents the 95% CI of average activity. Analysis of activity fragmentation (E) under LD12/12 (upper panel) and DD (lower panel) regimes of wild type (light gray circles) and *Hrh3* ^*-/-*^ (black circles) animals.

Under LD12/12 cycle *Hrh1* ^*-/-*^ mice had a prominent diurnal activity rhythm (Fig 1A and 1B, S1 File, Fig 1A and 1B), with a main period 24±0.07 h (p<0.01, χ^2^-periodogram, Fig 1C, upper panel) and were completely entrained to the LD 12/12 cycle. In DD, 11 out of 13 *Hrh1* ^*-/-*^ animals had free-running activity with the main period 23.71±0.09 h (p<0.001, χ^2^-periodogram, Fig 1C, lower panel), which was indistinguishable (n = 24, p = 0.31, Student's t-test) from wild-type mice—23.67±0.18 h, whereas two of the *Hrh1* ^*-/-*^ mice had no statistically significant periodicity (p>0.05, χ^2^-periodogram). We were not able to find any differences between acrophases and amplitudes of locomotor activity between the *Hrh1* ^*+/+*^ and the *Hrh1* ^*-/-*^ mice under the LD12/12 regime (Z-test, p = 0.345 and p = 0.328 respectively, Fig 1D) or the DD regime (Z-test, p = 0.363 and p = 0.376 respectively; Fig 1D; all the animals were included in analysis) regimes. Linear mixed model analysis of the effects of genotype, daytime and illumination regime on fragmentation of locomotor activity revealed strong effect of interaction between daytime and the regime (*F*_1,71_ = 10.062, p = 0.022, maximum likelihood) whereas neither interaction nor main effects of genotype were significant (*F*_1,71_ = 0.06, p = 0.797, maximum likelihood, Fig 1E). A weekly activity bursts (Figs 1A, 1B, 2A and 2B) for all mice corresponded to a bedding change.

The *Hrh3* ^*-/-*^ strain under the LD12/12 regime was completely entrained to a light-dark cycle with a main period 23.98±0.06 h (p<0.001, χ^2^-periodogram, Fig 2A and 2B). Under LD12/12 condition the amplitude and mesor of the knockout group were not significantly different from those of the wild type siblings (Z-test, p = 0.288 and p = 0.336 respectively, Fig 2D). Under DD regime *Hrh3* ^*-/-*^ animals had free-running activity with the main period 23.69±0.7 h (p<0.01, χ^2^-periodogram, Fig 2C, lower panel), which was similar to wild-type mice—23.8±0.15 h (n = 28, p = 0.56, Student's t-test). The amplitude and mesor were dramatically reduced compared to those of the wild type animals (Z-test, p = 0.007 and p<0.001 respectively, Fig 2D) resulting in increased variability in period estimation. Linear mixed model analysis of effects of genotype, daytime and illumination regime on fragmentation of locomotor activity revealed strong effect of interaction between daytime and the regime (*F*_1,71_ = 146.05, p<0.001, maximum likelihood) whereas neither interaction nor main effects of genotype were significant (*F*_1,71_ = 0.018, p = 0.8944, maximum likelihood, Fig 2E).

### Expression of *Per1*, *Per2* and *Bmal*1 genes

**Fig 3:**
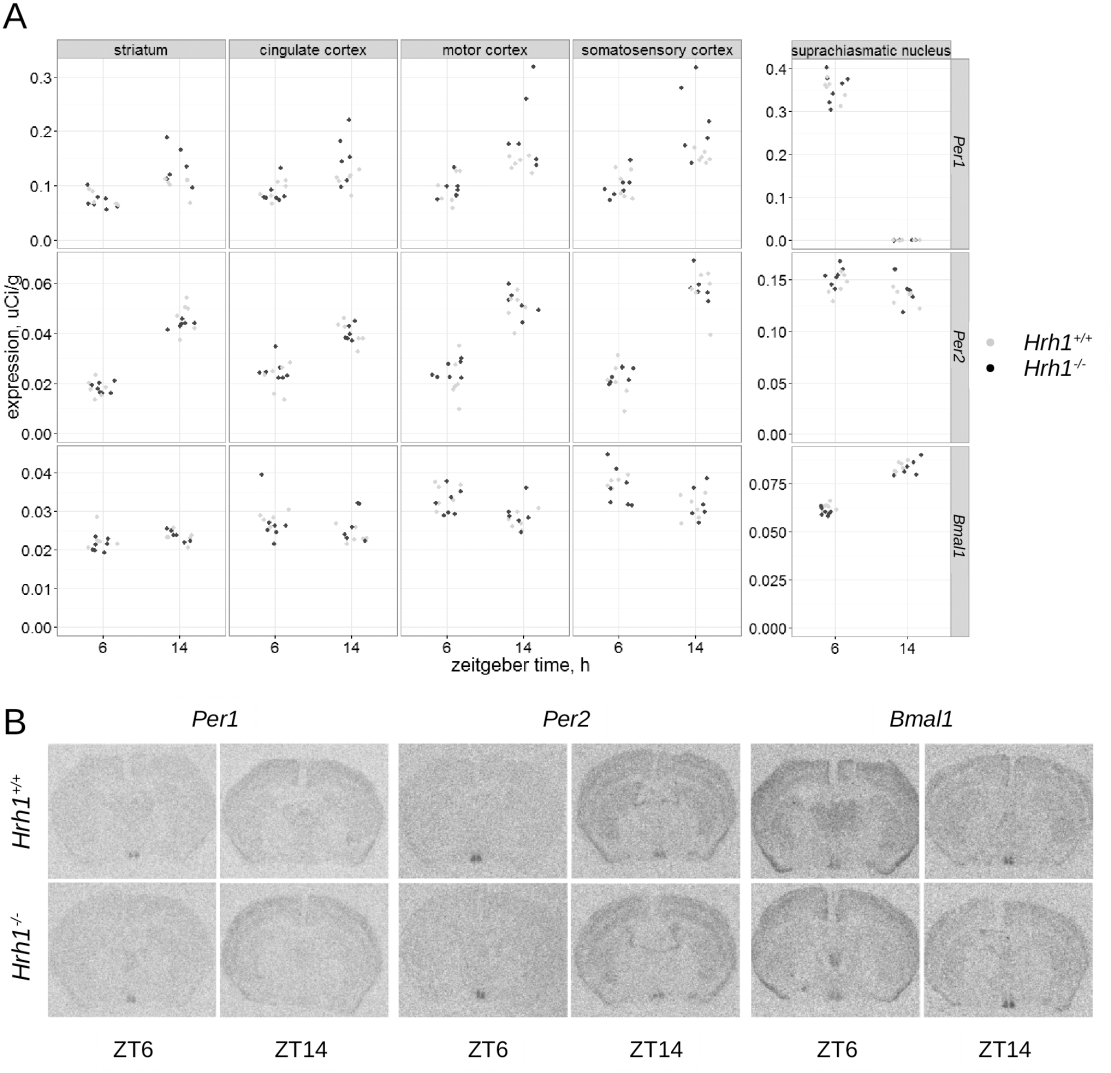
(A) Diurnal expression of *Per1* (upper panel), *Per2* (middle panel) and *Bmal1* (lower panel), genes. The expression was analyzed in the striatum, cingulate, motor and somatosensory areas of cerebral cortex and the SCN of wild type (light gray dots, n = 6) and *Hrh1* ^*-/-*^ (black dots, n = 7) mice at ZT6 and ZT14 housed under LD12/12 cycle. (B) Corresponding autoradiograms.

**Fig 4:**
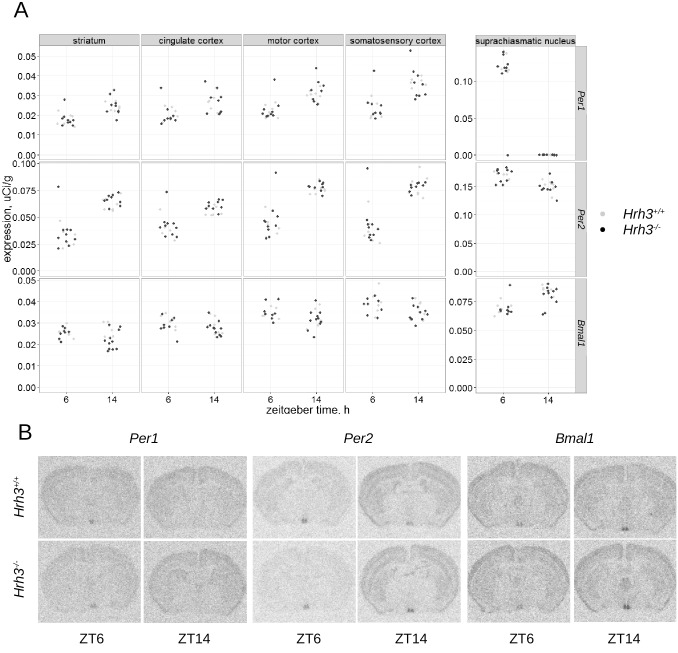
(A) Diurnal expression of *Per1* (upper panel), *Per2* (middle panel) and *Bmal1* (lower panel), genes. The expression was analyzed in the striatum, cingulate, motor and somatosensory areas of cerebral cortex and the SCN of the wild type (light gray dots, n = 6) and the *Hrh3* ^*-/-*^ (black dots, n = 10) mice at ZT6 and ZT14 housed under LD12/12 cycle. (B) Corresponding autoradiograms.

The expression of *Per1*, *Per2* and *Bmal1* genes in *Hrh1* ^-/-^ (Fig 3), *Hrh3* ^-/-^ (Fig 4) and wild type mice was assessed at ZT6 and ZT14 in the cingulate, motor and somatosensory areas of cerebral cortex, striatum and SCN by radioactive *in situ* hybridization. Linear mixed-model analysis followed by the leave-one-out test revealed no significant difference (p = 0.085, maximum likelihood estimation) of interaction between genotype, zeitgeber time and brain structure on expression of *Per1* gene in *Hrh1* ^-/-^ animals. No other differences in expression of these genes between knockout and wild type animals were found.

## Discussion

It has been shown previously that the lack of histamine alters free-running rhythms of spontaneous locomotion and expression of core clock genes in the striatum and several areas of cerebral cortex [16]. Therefore, the goal of the current study was to estimate the role of the *Hrh1* and *Hrh3* receptors in the regulation of circadian rhythm of locomotor activity and in expression of *Per1*, *Per2* and *Bmal1* genes.

We assessed spontaneous locomotion of *Hrh3* ^*-/-*^ mice under the DD regime and found a 50% decrease in this parameter compared with the wild type mice. Several studies on *Hrh3* ^-/-^ mice found diminished spontaneous locomotion [19,25], voluntary movement, body temperature [19] and time spent awake during the scotophase but not the photophase compared to the corresponding activity parameters for wild type animals. Toyota and colleagues [19] accessed the voluntary (running wheel) activity of *Hrh3* knockout mice under a DD regime and found its reduction by 25%, although its circadian rhythm was preserved. This finding is similar to that described for *Hdc* ^/-^ animals whose wheel running activity rhythm was significantly blunted under both LD and DD conditions [16]. This reduction in activity can be explained by histamine-dependent involvement of *Hrh3* in voluntary activities and/or overall reduction of locomotion during the scotophase, being caused by low histamine levels, which is a typical for *Hrh3* ^*-/-*^ mice [19]. Therefore, our results, combined with running wheel activity data described elsewhere [19] can be interpreted that both voluntary and also involuntary activities were indeed affected by the absence of functional *Hrh3*.

It is important to note that *Hrh3* is constitutively active [26] and can serve as both presynaptic and postsynaptic heteroreceptor, which participates in the control of release and actions of other neurotransmitters [27–29]. Thus, whether the phenotype observed in our present study was caused by altered histamine-mediated signaling or impaired modulation of other neurotransmitter systems remains to be investigated.

The proposition that *Hrh1* participates in the transduction of circadian information in mammals has gained support from findings of intense ^3^H-pyrilamine binding in the SCN area of mice [18] and rats [30]. The *Hrh1* antagonist pyrilamine blocked histamine-induced depolarization of cultured SCN neurons [31], but see [32], presumably via Ca_V_1.3-dependent route [33]. Rats housed under a LD12/12 regime and chronically administered with pyrilamine had an attenuated amplitude of locomotor activity [34]. In addition, several studies on *Hrh1* ^-/-^ mice reported altered feeding rhythms [35] and minor decrease in overall locomotor activity [18,36], but see [37].

It is worth noting that none of the aforementioned studies assessed the parameters in question under DD conditions. Therefore, even if *Hrh1* had indeed participated in the mediation of circadian information, then this effect would probably be masked by light and therefore would not have exhibited under such experimental designs. We, however, found that *Hrh1* ^-/-^ mice housed under DD had a free-running locomotor activity period similar to that of the wild type animals, and that daily changes in transcription of *Per1*, *Per2* and *Bmal1* genes in all examined brain structures were also similar to those of their wild type siblings.

The role of *Hrh2* in circadian regulation should be discussed although this was not addressed in the current study. Pharmacological inhibition of *Hrh2* by cimetidine was reported to lead to occasional potentiation of the effect of histamine on SCN neurons in rat brain slice preparations [31,32], but this treatment did not cause any phase shifts, when administered to hamsters [38]. Similarly, the brain-penetrating *Hrh2* antagonist zolantidine had no effect on light-induced phase shift as shown for hamsters, or the lengths or distributions of NREM, REM and wakefulness states as shown on rats [39]. Taking into account these reports, *Hrh2* appears therefore to be unlikely to play a major role in the regulation of the circadian system.

There are some methodological considerations regarding this study that should be addressed. Gene expression was examined under the LD12/12 rhythm, therefore the masking effect of the light may confound the measurement of the expression. The choice of sampling time (ZT6 and ZT14) was based on those used in previous studies that identified minima and maxima of expression for selected genes in multiple brain areas. These areas include the following: *Per1* expression in SCN [40,41], cerebral cortex and striatum [16]; *Per2* in SCN [42], cerebral cortex and striatum [6,16], *Bmal1* in SCN and cerebral cortex [43,44]. Nevertheless, this choice does not rule out the possibility of these being sub-optimal sampling times for knockout models. The effect of *Hrh3* ^*-/-*^ genotype on spontaneous locomotion was observed under the DD regime therefore further studies are necessary to reveal the extent of the expression of tested clock genes affected by these circumstances.

We used the classical constitutive knockout preparations, whereby the gene modification was present through the entire development of the animal. Thus, we cannot rule out the possibility of compensatory changes that might have occurred that would confound measured parameters in such genotypes. Such phenomena would introduce apparent contradictions to previously published results where other models were studied. The use of inducible knockouts could circumvent this problem.

Although the *Hdc* ^-/-^ mice have abnormally long free-running activity rhythm and lack periodic expression of *Per1* and *Per2* genes in the cerebral cortex and the striatum [16], the *Hrh1* ^-/-^ and *Hrh3* ^-/-^ strains had no change in the period length of the activity rhythm or expression of *Per* genes. Several studies reported discrepancies between histamine-deficient and histamine-receptor-deficient phenotypes. For example, light-induced phase shifts in hamsters were reduced by administering α-fluoromethylhistidine an inhibitor of *Hdc* [45], but this effect could not be blocked by *Hrh1*, *Hrh2*- or *Hrh3*-antagonists [36]. Saligrama et al., [46] demonstrated in 2013 that severe experimental allergic encephalomyelitis could be induced in *Hdc* ^-/-^ mice, whereas quadruple histamine receptor knockout animals were indistinguishable from wild type mice. If all effects of histamine are mediated through the four known G protein-coupled receptors, then the knocking out all receptors could be expected to reveal the same phenotype as a lack of histidine decarboxylase, which removes histamine synthesis. These results raise the possibility that histamine may exert its effects not only through its canonical receptors but also through other, currently unknown, mechanisms.

In conclusion, the *Hrh1* ^*-/-*^ model induced no detectable effects on the circadian locomotor rhythm, although *Hrh3* ^*-/-*^ caused a substantial reduction of free-running activity rhythm amplitude and therefore this receptor could be involved in its regulation. Under symmetric 24 h light-dark cycle the constitutive knocking out of the *Hrh1* and *Hrh3* receptors did not affect the expression patterns of the core clock genes in any of the studied brain structures. Therefore future work under constant darkness conditions could reveal whether the altered amplitude of locomotor activity rhythms in the *Hrh3* ^-/-^ mice is due to a circadian clock defect or to an altered regulation of the locomotor activity itself, downstream of the clock.

## Supporting Information

S1 File Data used to prepare the figures. Each subject in the study may have different subject number depending on statistical test.
(XLSX)
